# Multielemental Analysis of Bee Pollen, Propolis, and Royal Jelly Collected in West-Central Poland

**DOI:** 10.3390/molecules26092415

**Published:** 2021-04-21

**Authors:** Eliza Matuszewska, Agnieszka Klupczynska, Krzysztof Maciołek, Zenon J. Kokot, Jan Matysiak

**Affiliations:** 1 Department of Inorganic and Analytical Chemistry, Poznan University of Medical Sciences, Grunwaldzka 6 Street, 60-780 Poznań, Poland; eliza.matuszewska@ump.edu.pl (E.M.); aklupczynska@ump.edu.pl (A.K.); 2 Aquanet Laboratory Ltd., 126 Dolna Wilda Street, 61-492 Poznań, Poland; krzysztof.maciolek@aquanet-laboratorium.pl; 3 Faculty of Health Sciences, Calisia University, 13 Street, 62-800 Kalisz, Poland; z.kokot@akademiakaliska.edu.pl

**Keywords:** bee products, multielemental analysis, biomonitoring, ICP-MS, ICP-OES, inorganic contaminants, heavy metals

## Abstract

Beehive products possess nutritional value and health-promoting properties and are recommended as so-called “superfoods”. However, because of their natural origin, they may contain relevant elemental contaminants. Therefore, to assess the quality of bee products, we examined concentrations of a broad range of 24 selected elements in propolis, bee pollen, and royal jelly. The quantitative analyses were performed with inductively coupled plasma-mass spectrometry (ICP-MS) and inductively coupled plasma optical emission spectrometry (ICP-OES) techniques. The results of our research indicate that bee products contain essential macronutrients (i.e., K, P, and S) and micronutrients (i.e., Zn and Fe) in concentrations depending on the products’ type. However, the presence of toxic heavy metals makes it necessary to test the quality of bee products before using them as dietary supplements. Bearing in mind that bee products are highly heterogenous and, depending on the environmental factors, differ in their elemental content, it is necessary to develop standards regulating the acceptable levels of inorganic pollutants. Furthermore, since bees and their products are considered to be an effective biomonitoring tool, our results may reflect the environment’s condition in west-central Poland, affecting the health and well-being of both humans and bees.

## 1. Introduction

Macroelements and trace elements (including heavy metals) play an essential role in human nutrition. Since they are involved in many biochemical processes, they are crucial for life processes [1,2]. Moreover, minerals are components of biological structures, i.e., bones, nerves, and muscles. They are also included in enzymes, hormones, and pigments, such as oxygen-carrying hemoglobin [3]. Although a precise distinction between macronutrients and micronutrients is still under discussion, at least 23 of the minerals are defined as essential for humans [4,5]. They are the macronutrients: sodium (Na), potassium (K), magnesium (Mg), calcium (Ca), chlorine (Cl), phosphorus (P), and sulfur (S), and micronutrients: manganese (Mn), iron (Fe), copper (Cu), zinc (Zn), selenium (Se), cobalt (Co), molybdenum (Mo), and iodine (I). Some other elements are also discussed to be considered essential, such as vanadium (V), nickel (Ni), and silicon (Si) [4].

Deficiencies in macroelements and microelements cause serious health problems [6,7]. On the other hand, if consumed at excessively high levels for a long time, minerals can be toxic and trigger adverse effects [8]. They may disrupt the metabolic pathways by accumulating in the body, replacing the appropriate ions, or binding to enzymes responsible for controlling the metabolic reactions [9]. Moreover, by increasing the production of reactive oxygen species (ROS), mineral elements may also cause cancer, degenerative diseases, and damage to the central nervous system [10,11]. Therefore, consumers should be aware of the mineral content of foods. However, not all food products have been thoroughly investigated yet, including bee products.

Bee products are complex natural mixtures produced by a honey bee (*Apis mellifera*). The products are rich in nutrients and biologically active compounds, such as carbohydrates, proteins, amino acids, lipids, vitamins, phenolics, and minerals [12,13]. Due to their health-supporting properties, bee products are willingly used as dietary supplements within the branch of alternative medicine called apitherapy [14]. The most recognizable bee products are honey, bee venom, propolis, bee pollen, and royal jelly. In this research, we examined the selected minerals’ levels in three of them: bee pollen, propolis, and royal jelly.

Bee pollen is a mixture of flower pollen from different species gathered by forager bees, formed into pellets with nectar and secretions of bees’ salivary glands. In Poland, this beehive product’s main plant sources are *Brassica napus*, *Taraxacum officinale*, *Robinia pseudoacacia*, *Tilia cordata,* and *Trifolium repens*. Ethanol extract of Polish bee pollen was reported to be rich in flavonoids and polyphenols as well as responsible for anti-atherogenic activity [15]. Moreover, due to its nutritional and energetic properties, bee pollen supplementation is recommended for people leading active lifestyles and during recuperation [16]. Propolis, a resinous product with an aromatic smell, is produced by mixing bees’ saliva, beeswax, and pollen with secretions collected from plants [17]. In Poland’s temperate climate, its primary plant source is *Populus spp*., *Betula verucosa*, *Pinus sylvestris*, *Aesculus hippocastanum,* and *Acer pseudoplatanus* [18]. The botanical origin determines the main biologically active constituents of Polish propolis: flavonoids, phenolic acids, and esters. These compounds are responsible for bacteriostatic, antifungal, antioxidative, and antiproliferative properties [18,19,20,21]. Royal jelly is a milky secretion of worker bees’ salivary glands [22]. Unlike bee pollen and propolis, it does not contain plant tissues. In addition to water, royal jelly contains mainly sugars, proteins (including enzymes), amino acids, and lipids [12]. This bee product is becoming increasingly popular, as its antibacterial, anti-inflammatory, antioxidative, and even antitumor properties have been reported [22,23,24,25].

Bee pollen, propolis, and royal jelly are recommended as so-called “superfoods”, meaning foods of rich nutrient content having favorable effects on human health [26]. Therefore, they should be of the highest quality. However, the challenge is a variability of the composition of bee products, depending on the geographical region, climate, and season [16,27,28,29]. Differences may involve many groups of nutrients, including mineral contents [30,31]. Since excessive element intake may lead to adverse effects, the concentrations (along with the acceptable levels) of heavy metals and other mineral contaminants in bee products should be precisely defined.

The multielemental composition of bee products may also reflect environmental pollution, affecting both humans and bees. A continuous decline in the honeybee population has been reported in industrialized countries [32]. Several factors are supposed to contribute to honeybee declines, such as pests and diseases, global warming, and environmental pollutants associated with industrial and agricultural human activity, including heavy metals [32,33]. Due to the many vital roles of honeybees in the environment and agriculture, their health has become a public concern. Thus, it is imperative to assess the pollution to which bees are exposed in their natural habitat.

Therefore, to assess the safety of bee products for humans and bees, in this study, we examined concentrations of 24 selected elements (including macronutrients and micronutrients) in propolis, bee pollen, and royal jelly. We decided not to analyze bee venom, which was thoroughly examined in our previous study [34], and honey, which other researchers extensively studied [35,36,37,38]. The bee product samples used in this study were collected in Poland (Greater Poland region) in 2018 and 2019. The quantitative analyses were performed with inductively coupled plasma-mass spectrometry (ICP-MS) and inductively coupled plasma optical emission spectrometry (ICP-OES) techniques. Our research results contributed to assessing bees’ exposure to various chemicals and evaluating their products’ quality.

## 2. Results

Levels of 24 chemical elements were determined in all samples of bee products. We measured one of the broadest spectra of chemical elements (including micro- and macronutrients, heavy metals, and trace elements) in bee products compared to the available literature [39,40,41,42,43,44,45,46,47,48,49]. In this study, we examined various beehive matrices (bee pollen, propolis, and royal jelly) collected over two years from the same area. The majority of studies on the determination of elements (mainly heavy metals) in bee products included one selected product collected from different locations [31,39,46,48,49,50]. The analysis of different beehive products derived from the same area allowed us to compare the levels of inorganic contaminants between them and evaluate their role as bioindicators. On the other hand, the obtained results allowed comparing bee products in terms of micronutrient and macronutrient content. For the studied products, the determined concentrations of minerals, along with mean, standard deviations (SD), and relative standard deviation (%RSD) values, calculated for each year separately and all samples in total, are presented in Table 1 and Table 2.

The highest content in bee pollen was measured for K (mean = 4233.33 mg/kg), P (mean = 4050.00 mg/kg), and S (mean = 2383.33 mg/kg). These elements are classified as macroelements, for which the dietary requirement exceeds 100 mg per day. On the other hand, the lowest concentration in the macronutrient group in bee pollen was found for Na (mean = 25.17 mg/kg). Concerning micronutrients, the highest content in bee pollen was recorded for Zn (mean = 31.3 mg/kg), Fe (mean = 114.5 mg/kg), and Mn (mean = 25.0 mg/kg), and the lowest was found for Co (mean = 0.038 mg/kg). However, a relatively high Al content was measured in a group of toxic metals in bee pollen (mean = 26.13 mg/kg). On the other hand, Sb (mean = 0.009 mg/kg) and As (mean = 0.02 mg/kg) levels were the lowest among the bee pollen’s trace elements.

In propolis, the highest content among macronutrients was determined for K (mean = 706.67 mg/kg), Ca (mean = 373.33 mg/kg), and S (mean = 225.0 mg/kg). However, the concentrations of these elements in propolis were much lower than in bee pollen. The macroelement with the lowest content in propolis, similar to bee pollen, was Na (mean = 22.67 mg/kg). Among microelements, similar to bee pollen, the highest content was found for Zn (mean = 13.67 mg/kg). However, the concentration of Zn in bee pollen was over two times higher than in propolis. The micronutrient with the second-highest content in propolis was Fe (mean = 49.17 mg/kg) followed with Mn (mean = 7.2 mg/kg), but its concentration was more than three times lower than in bee pollen. The microelement with the lowest content in propolis was Se (mean = 0.047 mg/kg). Similar to bee pollen, the high Al content was measured in propolis (mean = 93.0 mg/kg), and this concentration was more than three times higher than in bee pollen. Among trace elements in propolis, Ag was determined at the lowest level (mean = 0.006 mg/kg). Summing up, mean levels of Mn, Ni, Co, Zn, Ag, Cd, Mo, Na, Mg, K, Ca, P, and S were higher in bee pollen than in propolis.

Analysis of bee product samples collected from the same location but in different months and years allowed us to estimate the variability in elemental composition caused by the date of sample collection. We observed quite substantial differences in the levels of individual elements in the beehive products between months and years of sample collection (Table 1 and Table 2). Among all determined elements in bee pollen, Cd, Al, and Ag showed the greatest variation in concentration between years (%RSD > 80%), whereas K, P, S, and Zn were present in a similar concentration throughout all analyzed years (%RSD < 20%). High %RSD values calculated for Ag and Cd can result from a very low level of that element determined in pollen samples. However, Al, Cd, and Ag constitute metals of anthropogenic origin. Thus, variation in their concentrations observed in bee pollen samples throughout the analyzed years can also reflect changes in the degree of pollution of the environment surrounding the apiary. The content of chemical elements determined in propolis samples did not vary so substantially as in the case of pollen, and the calculated %RSD values were noticeably lower (Table 2). The only exception was Ag, whose concentration occurred below the quantification limit (QL) in part of the samples.

**Figure 1:**
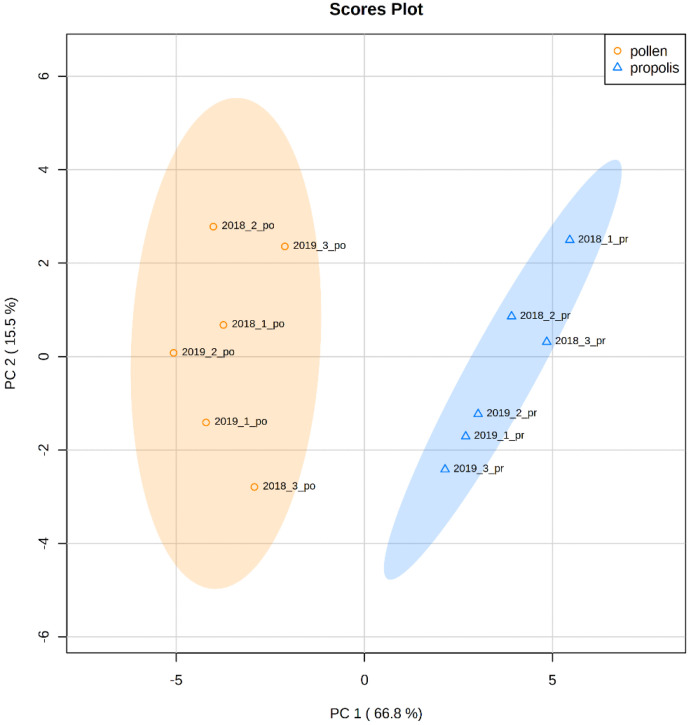
The principal component analysis (PCA) score plot using the first two principal components derived from elements’ determination in two beehive products: bee pollen (orange circles; *n* = 6) and propolis (blue triangles; *n* = 6).

To better assess variability in the elemental composition of different beehive products and different years of collection, univariate and multivariate statistical analyses were performed. Univariate statistical tests showed no significant differences in the elemental composition between bee pollen samples collected in two different years (Table 3). In the comparison of the elemental composition of propolis samples collected in two different years, significant differences were observed for six elements (Al, Ba, Cr, Fe, Sb, and Si). On the other hand, most of the observed differences in the levels of chemical elements determined in two various beehive products (pollen and propolis) were statistically significant. It should be emphasized that all studied samples were collected from the same location and during the same season; thus, the observed differences are not related to geographical region, climate, and season but are more associated with the process of the production of these bee products. Only for Cd, Na, Ni, and Se were differences not significant; thus, we can conclude that those elements occur at comparable levels in bee pollen and propolis. To better visualize how distinct the content of elements between pollen and propolis is, PCA analysis was conducted. A clear separation of the bee pollen samples and propolis samples was attained on the score plot (Figure 1). The results of PCA indicate that the differences in the elemental composition of pollen and propolis were strong enough to cause grouping of the samples according to the type of beehive products. It should be noted that the results of multivariate statistical analysis are in line with the results of univariate tests. Figure 1 demonstrates that differences in elements’ concentrations are more pronounced between two beehive products than between years of collection of these products. Moreover, it is visible that propolis samples collected in 2018 are separated from propolis samples collected in 2019, whereas no grouping is visible in the case of pollen samples.

Additionally, we analyzed levels of elements in the royal jelly sample collected in 2019. The concentrations of chemical elements measured in royal jelly compared to mean values calculated for the bee pollen and propolis samples are shown in Table 4. Among the macroelements, the highest content in royal jelly was measured for P (1700 mg/kg) and S (1200 mg/kg), whereas the lowest concentration was found for Ca (35 mg/kg). Concerning microelements, the highest content in royal jelly, similarly to bee pollen and propolis, was measured for Zn (21 mg/kg), and the lowest was measured for Co (0.003 mg/kg), as in bee pollen. Among the other elements, the highest concentration was found for Al and the lowest was found for V (0.001 mg/kg). In general, most of the elements’ levels were lower in royal jelly than in propolis or bee pollen. The exception is the content of Na, which was the highest in the royal jelly sample. Moreover, Cu, Zn, Ag, Mg, K, P, and S were at higher levels in royal jelly than in the propolis.

## 3. Discussion

Beehive products are a rich source of nutrients and biologically active compounds. As reported in the available literature, they contain macro- and microelements essential for the human body’s proper functioning [50,51,52,53]. In this study, we examined the concentrations of several macroelements, including Ca, K, Mg, Na, P, and S, and microelements, including Co, Cu, Fe, Mn, Mo, Se, and Zn in Polish bee pollen, propolis, and royal jelly.

Our study results indicate that bee pollen and royal jelly contain large amounts of K, P, and S. Additionally, bee pollen is rich in Ca. The same macronutrients are present at the highest level in propolis, but the concentrations are lower than in bee pollen and royal jelly. K, P, Ca, and S play a vital role in human health. K ensures energy metabolism, membrane transport, and cardiac contraction. This element is commonly found in food, so severe deficiencies are virtually non-existent [54]. However, the intake of K in modern diets is well below the current recommended nutritional requirements, which, according to the World Health Organization (WHO), is 3510 mg/day [55,56]. A sufficient intake of K is particularly important because K prevents cardiovascular disease and hypertension, kidney stones, and osteoporosis [55,57]. The second element, P, is a vital intracellular anion. It participates in various metabolic processes and serves as a component of cell membranes. In the body, it is usually found in the form of phosphate (PO_4_^3-^). P is essential in a variety of key biological molecules, including adenosine triphosphate (ATP) and 2,3-diphos-phoglycerate (2,3-DPG) [3,58]. Along with Ca, P is crucial for teeth and bone development [59]. Ca plays also a significant role in cell signaling and biochemical processes [60]. Disruption of Ca-P homeostasis leads to skeletal and cardiovascular disorders [61]. Therefore, the intake of these nutrients should be balanced. However, due to the use of phosphorus-based food additives, the phosphorus content of food has increased significantly [62,63] and in the United States, it far exceeds current recommendations for daily intake [64]. On the other hand, dietary intakes of the least abundant element, S, have decreased as a consequence of modern agricultural practice [65]. S is a component of important molecules, such as proteins, enzymes, and vitamins [66]. Hence, because of the high S, K, Ca and other essential macronutrients content proven in our study, bee products’ consumption is beneficial for the human body.

Among determined micronutrients, we found Zn to be the most abundant in bee pollen, royal jelly, and propolis (Table 3). Zn is essential in all types of human tissues. It serves as a component or an activator of various enzymes involved in more than 300 enzymatic reactions [67]. Zn supplementation may prevent or facilitate the treatment of depressive illnesses, diarrhea, and, by enhancing immune responses, prevent pneumonia and viral diseases [68,69,70,71]. In addition to Zn, we measured relatively high Fe and Mn levels in all analyzed bee products. In the available literature, it has been already proven that bee pollen is an excellent source of Zn and Fe [42,72]. Fe possesses an essential role in several enzymatic functions, including oxygen transport and oxidative phosphorylation [73]. Fe deficiency, which is the leading cause of anemia worldwide, affects nearly one-third of the population [74]. Moreover, the deficit of Fe in infants results in learning and memory deficits [75]. Mn, similar to Fe, is associated with the activation and synthesis of various enzymes. It is involved in cellular metabolism regulation, bone mineralization, blood clotting, and cellular protection from reactive oxygen species (ROS) [76]. For example, Mn deficiency may result in skeletal abnormalities, dermatitis, deafness, or infertility [77]. To summarize, our study proved that all studied honeybee products (bee pollen, propolis, royal jelly) should be considered as a valuable source of both macro- and micronutrients. However, the levels of particular elements varied significantly between different types of products (Table S2).

Although bee products are rich in selected micro- and macronutrients, Pohl et al. [78] have recently reported that not all minerals are bioaccessible for humans after pollen in vitro digestion. According to the results of that research, Ca and Mg are among the most accessible elements contained in bee products. However, the bioaccessibility of Zn, Fe, and Cu may be lower than 40%. In the case of micro- and macroelements, low absorption after digestion is unfavorable. However, although it has not been investigated, the low elements’ accessibility may also concern toxic heavy metals, and then, it is highly beneficial.

However, it should be kept in mind that elements occurring in natural products may have toxic effects on the body, even when consumed in small amounts. Taking into account the therapeutic and nourishing properties of bee products, it is important to know their contamination. Data from the available literature indicated that honeybee products are affected by environmental pollution [79]. However, there are scarce data that compare the inorganic contaminations (including heavy metals) between different bee products. All bee products contain potentially harmful trace elements and heavy metals, but our study results indicated significant differences in the degree of heavy metal contamination between them (Table S2). A clear distinction is observed, especially between the concentrations of heavy metals in the royal jelly compared to the bee pollen and propolis, which seem to be contaminated to a greater extent (Table 3). Among the elements with a potentially toxic effect on the human organism, Al was found at the highest levels in all studied beehive matrices. The highest Al concentration was observed in propolis, then bee pollen, and the lowest in royal jelly. Al is commonly found on Earth. However, due to anthropogenic activities, increasing Al content is observed in food [80]. Al accumulates in the brain, bones, kidney, and liver [81]. Prolonged exposure to even low levels of Al may lead to neurodegenerative disorders, such as Alzheimer’s disease [82]. Al can also interfere with some essential elements, such as Ca, by replacing it and thus affecting the bones’ mineralization [83].

Contamination of natural food is a serious problem, especially taking into account increasing environmental pollution. However, there is a lack of up-to-date regulations concerning bee products. Polish obsolete regulations specified only As, Cd, Cr, and Pb levels in honey (PN-88/A-77626: 1988), bee pollen (PN-R-78893: 1997), and propolis (PN-P-77627: 1997). Comparing our results to those regulations, we found that the studied samples of bee pollen fully met the requirements. However, levels of Cr and Pb in propolis were overdrawn. Excessive intake of Pb and Cr can lead to serious health consequences. Pb is toxic for children and adults, causing anemia, hypertension, cognitive deficits, immunodeficiency, infertility, bone and tooth development delays, vitamin D deficiency, and hepatic and renal effects [84,85]. The second element, Cr, although involved in human lipid and protein metabolism (in a form of Cr^3+^) [86], is also connected with several pathologies, including carcinogenic (in a form of Cr^6+^) [87,88]. Thus, finding these elements in propolis at excessive levels indicates that bee products must be tested before being released for consumption or medical use. However, proper regulations should be prepared, considering all potentially harmful trace elements and heavy metals. The results of our research may be the first step toward developing appropriate regulations.

The contaminants involved in bee products may arise both from beekeeping practice and from the environment. Trace elements and heavy metals can be transferred to honeybees and consequently to their products from all the environmental compartments (plants, soil, air, and water) in the areas adjacent to the hive [89,90]. The presence of toxic elements in bee pollen, propolis, and royal jelly is certainly associated with anthropogenic pollution around the apiaries. Hence, bees and their products can be used for effective environmental biomonitoring [91,92]. Road traffic and industry have a substantial impact on environmental pollution with trace elements and heavy metals. Elevated Cr content in the tested bee pollen samples may come from metal electroplating and metallurgical, paint, and leather tanning industries. The source of increased Pb levels can be metallurgical and glass industries. Additionally, Al can come from the cosmetic and pharmaceutical industries, Ni can come from the steel industry, and Cd can come from metal smelters [93,94]. Cd was also suggested to correlate with Pb contamination [46]. Moreover, heavy metal ions, such as Pb (II), Fe (III), Hg (II), Cu (II), and Cr (VI), are present in groundwater, which nowadays is one of the most severe environmental problems [95].

In addition to humans, bees are also affected by toxic heavy metal pollution. The data gathered from 2012 to 2014 in a pan-European epidemiological study on honeybee colony losses (EPILOBEE) revealed that bee colony mortality rates reached up to 36% [96]. A syndrome that is characterized by a sudden loss of worker bees in a colony has been named Colony Collapse Disorder [97]. A decline in bee colonies number affects all agricultural activities that rely on these pollinators’ activity [98]. The origin of this loss is undoubtedly an anthropogenic activity, generating environmental contamination with elevated levels of toxic elements and heavy metals. In the study of Polish forager bees, Roman [99] reported that elemental contaminations accumulate in the bees’ bodies to the degree depending on the industrialization of the region in which they live. Similar relationships were also observed by other researchers from different countries [100,101,102,103]. Bees’ exposition to elevated heavy metals contaminants may affect the expression of genes encoding mainly enzymes involved in the detoxification metabolism, which indicates the physiological response of bees toward environmental pollutions [104]. In addition, bees’ acetylcholinesterase levels have been suggested to correlate with inorganic contaminants [105]. Changes in enzymes expression may contribute to the disruption of homeostasis in bees, eventually causing their decline. Moreover, in bees exposed to heavy metals, alterations in feeding behavior have been observed [106]. Forager bees may reject the resources contaminated with some elements, such as Cu. On the other hand, they may prefer food moderately contaminated with Pb. That poses a significant risk to larvae, which are more sensitive to heavy metals than adult bees [79,106,107]. Feeding the larvae with contaminated resources may cause the accumulation of metals in their bodies, leading to an increased risk of brood survival decrease. A reduction in the number of bee colonies is a serious problem that requires immediate steps to prevent bees’ extinction. As a first step, it is necessary to assess the factors influencing bee losses, including the pollution to which bees are exposed in their natural habitat. Thus, this study’s results may contribute to broadening the knowledge of the quality of apiaries’ surroundings in west-central Poland. Analysis of the data can help in deciding on possible ways to prevent bee decline.

Since the raw plant materials that bees use to make pollen may be contaminated to varying degrees, trace elements and heavy metals content in bee products may depend on the type of bee product and the kind of plant the bees use to produce it. In our study, we detected significantly lower concentrations of most elements in royal jelly compared to bee pollen and propolis. This is probably due to the fact that bee pollen and propolis are made directly from the pollen and resins of the plants flowering in the area covered by forager bees. Plants tend to accumulate heavy metals absorbed from the environment, mainly from water [108]. On the other hand, royal jelly is a pure secretion of bees’ glands. Hence, the differences in elements content between royal jelly, bee pollen, and propolis may result from these products’ origins. In central-west Poland, where the samples were collected, the primary pollen source is annual or biennial plants. Thus, samples of bee pollen used in our study contained mainly pollen of *Phacelia tanacetifolia*, *Achillea millefolium*, *Anthriscus sylvestris,* and *Brassica napus*. These plants, living just for a short period, perfectly reflect the dynamic changes in the environment’s quality. Therefore, the results of our study may mirror the current environmental condition. Samples of the same bee product (collected from the same apiary) show some differences in contaminants levels, which are reflected in relatively high values of standard deviation (SD). Thus, it may be assumed that both bee pollen and propolis may be successfully used for biomonitoring.

Several reports presenting the elemental content in bee products collected in different regions of Earth can be found in the available literature. Temizer et al. [39] analyzed 20 elements in bee pollen collected in Turkey. In the study, the authors found the highest level of Fe in all pollen samples. In general, the Al, As, Cr, and Fe concentrations found in the reported research were higher than those in our study. On the other hand, Mn and Zn levels were higher in samples examined in our research (i.e., pollen samples of Polish origin). Turkish propolis was also analyzed by Altunatmaz et al. [40], who measured the concentrations of 12 chemical elements. The concentration values obtained in that study were in general higher than ours. Only levels of Ni, Pb, Si, and Zn were reported to be lower than in our research. The comparison of our results with data obtained by Formicki et al. [41], who analyzed Polish bee pollen and propolis, revealed that our studied samples contained, in general, lower levels of chemical elements. However, the authors of the above-mentioned article measured concentrations of only six elements: Cd, Ni, Pb, Fe, Mg, and Zn, whereas our study involved 24 elements. An explanation for the observed differences between beehive products may be the origin of the bee pollen and propolis used in the study. Although the samples in both studies were collected in Poland, our samples were collected in the Greater Poland Voivodeship in west-central Poland. The apiary where our samples were collected is located in Góry Złotnickie village. It is a typically agricultural region. There are fields within a radius of 20 km (mainly rapeseed, phacelia, and clusters of linden, acacia and fruit trees), as well as uncultivated lands and meadows. This area is several kilometers away from human settlements. The nearest bigger city (Kalisz, population c.a. 100,000) is about 20 km away from the apiary. In contrast, Formicki et al. used samples from Lesser Poland Voivodeship in southern Poland. The Lesser Poland region is an area of high industrial and agricultural activity. In this province, heavy metals are the most common and toxic contaminants [109]. Bee pollen was also analyzed by researchers from other countries, such as Serbia, New South Wales, and Greece (42–44). In these studies, the concentrations of the selected elements were generally higher than in Polish samples analyzed by us (see Supplementary Materials).

Polish propolis was also analyzed by Roman et al. [45] but only for Cd, Co, Pb, Zn, and As content. The concentration of the selected elements in that study was higher than in ours. This is also probably due to the location of the apiaries—Roman et al. analyzed propolis collected from a heavily industrialized region (Lower Silesia Voivodeship). Additionally, the apiaries were located in the area of Wrocław—a large provincial city. Comparisons of our study with the literature reports suggest that a higher pollutant content may be associated with higher industrial and agricultural activity. This observation supports the assumption that bee products are excellent matrices for biomonitoring.

Comparing our results with the elements’ levels measured in Brazilian propolis [46,47] indicated lower levels of both nutritional and toxic elements in samples of propolis collected in Poland. In addition, Polish propolis seems to be less contaminated than samples collected in Spain [48]. However, samples of Macedonian propolis [49] contained lower concentrations of the selected chemical elements than our beehive products. These notable discrepancies between the literature reports and our study prove that bee products are characterized by a high variability (see Supplementary Materials). For this reason, quality testing of bee products should be performed routinely.

In the available literature, there are very limited data reporting concentrations of chemical elements in royal jelly. Stocker et al. [50] analyzed royal jelly samples collected in France. The authors showed higher elemental concentrations in royal jelly than measured in our study. However, the correlation between the levels of individual elements in the study of Stocker et al. [50] and our research was comparable. Royal jelly, which is the pure secretion of bee glands and contains any components taken directly from plants or the environment, can also serve as a biomonitoring tool. Bees are inextricably linked to the natural environment. Therefore, both bee secretions and the bee’s whole body can reflect the degree of environmental pollution [110].

## 4. Materials and Methods

### 4.1. Sample Collection

Bee pollen, propolis, and royal jelly samples were harvested directly from hives of *Apis mellifera* bees in apiaries located in Góry Złotnickie village (N 51°87′504′′, E 18°12′431′′), Greater Poland Voivodeship in west-central Poland. Bee pollen and propolis samples were collected from May 2018 to July 2019 during the summer season. We analyzed three bee pollen samples collected in 2018 and three collected in 2019 as well as three propolis samples collected in 2018 and three in 2019. Moreover, pilot tests were conducted using a royal jelly sample collected in 2019. The samples were stored at – 80 °C in the darkness until analysis.

### 4.2. Sample Preparation

A microwave-assisted digestion method was used for sample preparation. Mineralization was conducted in closed vessels made of PTFE. The weighed honeybee products (0.25−0.75 g) were mixed with 10 mL of suprapure grade 65% nitric acid (Merck, Darmstadt, Germany). The mineralization temperature was set at 180 °C, and the duration was 20 min. After cooling, the minerals were quantitatively transferred to centrifuge tubes with a capacity of 50 mL, and the volume of the solution was adjusted to the mark with deionized water. The obtained solutions were filtered through 0.45 μm polypropylene syringe filters and diluted with deionized water in an appropriate ratio. The pilot tests and subsequent validation procedures demonstrated that the proposed method of mineralization is effective enough to convert the elements into a form enabling their quantitative determination.

### 4.3. Elemental Analysis

A quadrupole ICP-MS 7800 (Agilent Technologies, Tokyo, Japan) was used for the determination of heavy metals and trace elements (Ag, Al, As, Ba, Cd, Co, Cr, Cu, Mn, Mo, Ni, Pb, Sb, Se, V, and Zn) in the collected honeybee products. Additionally, ICP-OES experiments were performed to determine the main elements’ content (Ca, Fe, K, Mg, Na, P, S, and Si) in the studied beehive products. The assays were performed in accordance with the requirements of PN-EN ISO 17294-2: 2016-11 standard.

The methods used were validated in terms of linearity, the limit of detection, the limit of quantification, precision, and recovery. Calibration curves for each element were determined from seven calibration solutions analyzed in triplicate. Calibration solutions were prepared using commercial standard solutions (c = 1000 mg/kg, Merck Darmstadt, Germany), consistent with NIST standards, and the serial dilution method. The linearity of the calibration curves was found in the working range of the method for each of the analyzed elements. The instrumental limits of detection and quantification were estimated as three times and 10 times the standard deviation, respectively, from the measurements of a blank sample with the addition of a small amount of standard. Precision was determined by analyzing real samples representing three tested matrices (propolis, bee pollen, royal jelly). Each sample was analyzed in seven replicates (the entire analytical procedure starting from weighing the sample was conducted). Precision values expressed as coefficient of variation (%RSD) were determined for each type of matrix separately. In the case of unfortified samples, precision was evaluated only for analytes exceeding the limit of quantification in the tested samples. Due to some analytes’ low content in real samples, an additional determination of precision was performed for fortified samples. Precision (%RSD) was calculated based on three repetitions of each fortified sample. The analysis of the fortified matrices proved that the applied analytical technique is characterized by high precision (Supplementary Materials, Table S3). However, higher %RSD values were obtained based on analysis of unfortified samples. This could be partially explained by some elements’ content in the lower working range of the method (near the limit of quantification). Within the validation, recovery tests of the fortified samples were also performed. Honeybee products were fortified (before mineralization) at two concentration levels within the method’s working range. The percent recovery was calculated according to the equation:

%R = (conc. in fortified sample − conc. in unfortified sample)/(amount of standard added) * 100%

The obtained results were in line with generally accepted requirements, according to which the recovery values for pollutants present in amounts (m/m) below 0.1% should be within the range of 75–125%. The detailed values of the method parameters determined during validation are contained in Supplementary Materials in Table S3. To sum up, the performed validation experiments showed good linearity, satisfactory precision, and acceptable accuracy of the applied method.

Elemental analysis of the beehive products was preceded by an analysis of calibration solutions, based on which a calibration curve is prepared. The matrix of the calibration solutions corresponded to the matrix of the analyzed samples. In order to eliminate interferences of matrix origin, an internal standard method was used. The internal standard solution was introduced in the same amount to all analyzed solutions (calibration solutions, real samples, quality control samples) using a separate peristaltic pump line. A solution of Sc, Y and Tb was used as an internal standard. Polyatomic interferences were eliminated by using a crash chamber with the use of helium as a reaction gas.

### 4.4. Statistical Analysis

The statistical analyses were conducted using Statistica 13.0 (TIBCO Software Inc., CA, USA) and the MetaboAnalyst 5.0 [111]. In the first step of univariate statistical analyses, the Shapiro–Wilk test of normality was applied. Then, to assess the equality of variances, Levene’s test was used. Variables with normal distribution and equal variances were subjected to a t-test, whereas variables with normal distribution and not equal variances were subjected to Welch’s test. For the analysis of variables that were not normally distributed, the Mann–Whitney U test was used. In all tests, a *p*-value ≤ 0.05 was considered statistically significant. Additionally, in order to visualize the differences in the elemental composition of the analyzed bee products, a multivariate statistical analysis - principal component analysis (PCA) was conducted. Prior to PCA, autoscaling of the elements’ concentrations were performed.

## 5. Conclusions

In the current study, we analyzed one of the broadest ranges of chemical elements in the selected beehive products. Among the determined elements were macro- and micronutrients, trace elements, and heavy metals. Therefore, the obtained data contributed to assessing both nutritional value and levels of inorganic contamination of bee products collected in west-central Poland. Although bee pollen, propolis, and royal jelly are characterized by different micronutrient and macronutrient content, it is impossible to consider one product as the best dietary supplement. The choice of a bee product should be tailored to the condition whose treatment we want to support. It should be borne in mind that apart from micronutrients and macronutrients, bee products contain other valuable components, such as proteins, amino acids, lipids, vitamins, and minerals. However, the concentrations of heavy metals provide us information on the safety of using bee products. The levels of heavy metals measured in bee pollen and propolis were generally lower than in samples collected in Poland’s highly industrialized regions. These results suggest that due to the high variability of the contaminants of bee products from the same country, measurements should be performed routinely. Moreover, bee pollen samples analyzed in this study seem to be less contaminated than those collected in Turkey, and propolis examined in this research contained lower inorganic pollutants than collected in Brazil and Spain. These results may indicate better environmental quality in the vicinity of apiaries in west-central Poland. It may also be concluded that honeybees and their products may serve as sensitive bioindicators, reflecting the degree and dynamic changes of environmental pollution.

## Figures and Tables

**Table 1:** Levels of 24 selected elements measured in bee pollen along with mean, standard deviations (SD), and relative standard deviation (%RSD) values, calculated for each year separately and all samples in total; 1, 2, 3—samples collected in 6 May, 23 June and 8 July 2018, respectively; 4, 5, 6—samples collected in 16 May, 2 June and 20 July 2019, respectively. Values of mean and SD are expressed in mg/kg.

Element	1	2	3	Year 2018		4	5	6	Year 2019		Years 2018 and 2019	
	[mg/kg]			Mean + SD	%RSD	[mg/kg]			Mean + SD	%RSD	Mean + SD	%RSD
Ag	0.18	0.28	0.04	0.17 ± 0.12	72.33	0.05	0.09	0.05	0.06 ± 0.02	36.46	0.12 ± 0.10	83.54
Al	53.00	25.00	11.00	29.67 ± 21.39	72.09	7.20	9.60	51.00	22.60 ± 24.62	108.96	26.13 ± 20.99	80.31
As	0.03	0.03	0.01	0.02 ± 0.01	48.42	0.01	0.01	0.03	0.02 ± 0.01	79.52	0.03 ± 0.01	57.55
Ba	0.84	0.57	0.51	0.64 ± 0.18	27.47	0.25	0.65	1.00	0.63 ± 0.38	59.25	0.64 ± 0.26	41.17
Ca	1800.00	1500.00	450.00	1250.00 ± 708.87	56.71	1500.00	1500.00	680.00	1226.67 ± 473.47	38.59	1238.33 ± 539.27	43.55
Cd	0.02	0.11	0.03	0.05 ± 0.05	91.14	0.02	0.04	0.12	0.06 ± 0.06	96.25	0.06 ± 0.05	84.12
Co	0.03	0.05	0.02	0.03 ± 0.02	49.01	0.04	0.05	0.04	0.042 ± 0.003	7.16	0.038 ± 0.012	30.99
Cr	0.12	0.08	0.04	0.08 ± 0.04	50.00	0.04	0.03	0.11	0.06 ± 0.04	76.82	0.07 ± 0.04	57.54
Cu	5.30	5.30	2.40	4.33 ± 1.67	38.64	5.40	5.70	4.40	5.17 ± 0.68	13.17	4.75 ± 1.23	25.91
Fe	75.00	57.00	22.00	51.33 ± 26.95	52.50	24.00	49.00	68.00	47.00 ± 22.07	46.95	49.17 ± 22.16	45.07
K	4500.00	4100.00	4300.00	4300.00 ± 200.00	4.65	3500.00	4600.00	4400.00	4166.67 ± 585.95	14.06	4233.33 ± 398.33	9.41
Mg	1100.00	720.00	620.00	813.33 ± 253.25	31.15	980.00	1000.00	520.00	833.33 ± 271.54	32.58	823.33 ± 235.09	28.55
Mn	16.00	21.00	13.00	16.67 ± 4.04	24.25	22.00	62.00	16.00	33.33 ± 25.07	75.02	25.00 ± 18.44	73.76
Mo	0.31	0.29	0.16	0.25 ± 0.08	32.15	0.18	0.30	0.16	0.21 ± 0.08	35.49	0.23 ± 0.07	31.57
Na	25.00	32.00	14.00	23.67 ± 9.07	38.34	24.00	24.00	32.00	26.67 ± 4.62	17.32	25.17 ± 6.65	26.41
Ni	0.63	0.83	0.37	0.61 ± 0.23	37.81	0.66	0.74	0.67	0.69 ± 0.04	6.32	0.65 ± 0.15	23.81
P	4600.00	4100.00	3200.00	3966.67 ± 709.46	17.89	4300.00	4600.00	3500.00	4133.33 ± 568.62	13.76	4050.00 ± 582.24	14.38
Pb	0.17	0.19	0.12	0.16 ± 0.04	22.53	0.14	0.09	0.22	0.15 ± 0.07	45.35	0.15 ± 0.05	31.59
S	2700.00	2600.00	1,800.00	2366.67 ± 493.29	20.84	2600.00	2700.00	1900.00	2400.00 ± 435.89	18.16	2383.33 ± 416.73	17.49
Sb	0.007	0.013	0.005	0.008 ± 0.004	49.033	0.004	0.008	0.016	0.009 ± 0.006	64.58	0.009 ± 0.005	52.45
Se	0.04	0.06	0.04	0.05±0.01	32.46	0.02	0.03	0.09	0.05 ± 0.04	82.58	0.05 ± 0.03	56.12
Si	75.00	45.00	20.00	46.67 ± 27.54	59.01	8.50	22.00	71.00	33.83 ± 32.89	97.20	40.25 ± 28.02	69.63
V	0.11	0.05	0.03	0.06 ± 0.045	70.84	0.05	0.02	0.11	0.06 ± 0.05	76.48	0.06 ± 0.04	65.88
Zn	27.00	28.00	35.00	30.00 ± 4.36	14.53	26.00	31.00	41.00	32.67 ± 7.64	23.38	31.33 ± 5.75	18.35

**Table 2:** Levels of 24 selected elements measured in propolis along with mean, standard deviations (SD), and relative standard deviation (%RSD) values, calculated for each year separately and all samples in total; 1, 2, 3—samples collected in 19 May, 23 June and 8 July 2018, respectively; 4, 5, 6—samples collected in 19 May, 1 June and 29 June 2019, respectively. Values of mean and SD are expressed in mg/kg, QL—quantification limit.

Element	1	2	3	Year 2018		4	5	6	Year 2019		Years 2018 and 2019	
	[mg/kg]			Mean + SD	%RSD	[mg/kg]			Mean + SD	%RSD	Mean + SD	%RSD
Ag	<QL	0.01	<QL	0.003 ± 0.006	173.21	<QL	0.07	<QL	0.009 ± 0.016	173.21	0.009 ± 0.016	177.76
Al	140.00	120.00	120.00	126.67 ± 11.55	9.12	93.00	97.00	69.00	86.33 ± 15.14	17.54	86.33 ± 15.14	23.63
As	0.11	0.06	0.08	0.09 ± 0.03	26.98	0.06	0.06	0.05	0.056 ± 0.009	16.20	0.056 ± 0.01	32.40
Ba	3.90	2.60	2.90	3.13 ± 0.68	21.72	1.50	1.80	1.70	1.67 ± 0.15	9.17	1.67 ± 0.15	38.19
Ca	500.00	560.00	300.00	453.33 ± 136.14	30.03	300.00	220.00	360.00	293.33 ± 70.24	23.94	293.33 ± 70.24	34.99
Cd	0.05	0.04	0.03	0.043 ± 0.01	23.26	0.03	0.08	0.03	0.032 ± 0.006	19.52	0.032 ± 0.0184	25.56
Co	0.13	0.10	0.20	0.14 ± 0.05	35.80	0.10	0.10	0.08	0.09 ± 0.01	13.45	0.09 ± 0.01	36.95
Cr	0.69	0.72	0.54	0.65 ± 0.10	14.84	0.28	0.41	0.25	0.31 ± 0.09	27.14	0.31 ± 0.09	41.84
Cu	2.00	1.40	1.40	1.6 ± 0.35	21.65	3.00	1.10	0.98	1.69 ± 1.13	66.92	1.69 ± 1.13	45.62
Fe	150.00	160.00	120.00	143.33 ± 20.81	14.52	97.00	84.00	76.00	85.67 ± 10.60	12.37	85.67 ± 10.60	30.45
K	730.00	1100.00	470.00	766.67 ± 316.60	41.30	610.00	600.00	730.00	646.67 ± 72.34	11.19	646.67 ± 72.34	30.52
Mg	110.00	140.00	76.00	108.67 ± 32.02	29.47	100.00	64.00	110.00	91.33 ± 24.19	26.49	91.33 ± 24.19	27.10
Mn	7.90	8.30	6.40	7.533 ± 1.002	13.30	5.00	9.70	5.90	6.87 ± 2.49	36.33	6.87 ± 2.49	24.15
Mo	0.07	0.13	0.04	0.08 ± 0.05	58.10	0.04	0.05	0.06	0.05 ± 0.01	26.01	0.05 ± 0.01	51.43
Na	30.00	20.00	29.00	26.33 ± 5.51	20.91	23.00	20.00	14.00	19.00 ± 4.58	24.12	19.00 ± 4.58	26.71
Ni	0.53	0.86	0.47	0.62 ± 0.21	33.87	0.25	0.40	0.36	0.34 ± 0.08	23.07	0.37 ± 0.08	43.92
P	230.00	160.00	110.00	166.67 ± 60.28	36.17	300.00	190.00	210.00	233.33 ± 58.59	25.11	233.33 ± 58.59	32.25
Pb	1.00	0.68	0.74	0.817 ± 0.17	21.09	0.50	0.60	0.44	0.51 ± 0.08	15.75	0.51 ± 0.08	30.30
S	270.00	320.00	160.00	250 ± 81.86	32.74	180.00	200.00	220.00	200.00 ± 20.00	10.00	200.00 ± 20.00	26.63
Sb	0.04	0.04	0.05	0.042 ± 0.002	5.46	0.04	0.03	0.08	0.031 ± 0.005	17.07	0.031 ± 0.005	19.64
Se	0.08	0.05	0.04	0.057 ± 0.0	37.96	0.03	0.05	0.04	0.034 ± 0.008	19.87	0.038 ± 0.008	37.12
Si	170.00	160.00	140.00	156.677 ± 15.28	9.75	97.00	110.00	96.00	101.00 ± 7.81	7.73	101 ± 7.815	25.12
V	0.39	0.27	0.26	0.317 ± 0.07	23.59	0.20	0.22	0.15	0.19 ± 0.04	18.98	0.19 ± 0.04	32.95
Zn	19.00	12.00	11.00	14 ± 4.36	31.13	15.00	13.00	12.00	13.33 ± 1.53	11.46	13.33 ± 1.53	21.54

**Table 3:** Results of univariate tests performed to compare levels of chemical elements (*n* = 24) in two beehive products collected over two years from the same area: bee pollen and propolis. Bold text indicates a statistically significant difference with a *p*-value below 0.05.

Element	Pollen 2018 vs. 2019	Propolis 2018 vs. 2019	Pollen vs. Propolis
	*p* Value		
Ag	0.21856	NA *	**0.00508**
Al	0.72650	**0.02142**	**0.00015**
As	0.65361	0.10144	**0.00169**
Ba	0.97911	**0.02194**	**0.00430**
C	0.96446	0.14470	**0.01307**
Cd	0.93551	0.18140	0.81018
Co	0.34784	0.16963	**0.00537**
Cr	0.56601	**0.01054**	**0.00508**
Cu	0.46925	0.89808	**0.00824**
Fe	0.83994	**0.01289**	**0.00423**
K	0.72807	0.55699	**0.00508**
Mg	0.93016	0.49600	**0.00508**
Mn	0.31806	0.68966	**0.00508**
Mo	0.56704	0.38378	**0.00144**
Na	0.63668	0.15094	0.51126
Ni	0.58672	0.09351	0.13817
P	0.76675	0.24152	**0.00508**
Pb	0.81004	0.05422	**0.00123**
S	0.93433	0.36214	**0.00508**
Sb	0.82629	**0.02728**	**0.00003**
Se	1.00000	0.23406	0.92881
Si	0.63166	**0.00493**	**0.00052**
V	0.95890	0.06677	**0.00133**
Zn	0.62719	0.81490	**0.00021**

* NA—no statistical tests were performed because Ag concentration was determined only in one sample per year.

**Table 4:** Mean values of 24 selected chemical elements determined in bee pollen and propolis and measured levels of elements in the royal jelly sample. Values are expressed in mg/kg.

Chemical Element	Bee Pollen	Propolis	Royal Jelly			
Ag	0.12	0.01	0.09			**Legend:**
Al	26.13	106.50	1.00			
As	0.02	0.07	0.01			the highest
Ba	0.64	2.40	0.12			medium
Ca	1238.33	373.33	35.00			the lowest
Cd	0.06	0.04	0.002			
Co	0.04	0.12	0.003			
Cr	0.07	0.48	0.02			
Cu	4.75	1.65	4.20			
Fe	49.17	114.50	3.90			
K	4233.33	706.67	970.00			
Mg	823.33	100.00	120.00			
Mn	25.00	7.20	0.73			
Mo	0.23	0.07	0.05			
Na	25.17	22.67	41.00			
Ni	0.65	0.48	0.25			
P	4050.00	200.00	1700.00			
Pb	0.15	0.66	0.07			
S	2383.33	225.00	1200.00			
Sb	0.01	0.04	0.003			
Se	0.05	0.05	0.02			
Si	40.25	128.83	0.88			
V	0.06	0.25	0.001			
Zn	31.33	13.67	21.00			

## Data Availability

The data presented in this study contained within the article and supplementary materials.

## Supplementary Materials

The following are available online. Tables S1 and S2. Comparison of the selected elements’ levels in bee pollen to the available literature data; Table S3. Performance characteristics of the analytical method used; Figures S1–S17: Bar charts visualizing the differences between levels (mean±SD) of the selected heavy metals measured in bee pollen and propolis in our study and by other research teams.

## Sample Availability

Not available.
