# Supplementary material for: Multielemental Analysis of Bee Pollen, Propolis, and Royal Jelly Collected in West-Central Poland
Source: Molecules. 2021 Apr 21;26(9):2415. doi: 10.3390/molecules26092415 (PMC8122449; doi:10.3390/molecules26092415)
Supplement: Supplementary file 1 [file molecules-26-02415-s001.zip › molecules-1178358-supplementary.pdf]

Multielemental analysis of bee pollen, propolis and royal jelly collected in west-central Poland

Eliza Matuszewska[1], Agnieszka Klupczynska[1], Krzysztof Maciołek[2], Zenon J. Kokot[3] and Jan Matysiak[1,*]

[1]Department of Inorganic and Analytical Chemistry, Poznan University of Medical Sciences, Grunwaldzka 6 Street, 60-780 Poznań, Poland;

[2]Aquanet Laboratory Ltd., 126 Dolna Wilda Street, 61-492 Poznań, Poland;

[3]Faculty of Health Sciences, Calisia University - Kalisz, Poland, Kaszubska 13 Street, 62-800 Kalisz, Poland;

*Correspondence: jmatysiak@ump.edu.pl

Supplementary Materials

Content:

Table S1. Levels (mean±SD) of the selected chemical elements in bee pollen compared to the available literature data.

Table S2. Levels (mean±SD) of the selected chemical elements in propolis compared to the available literature data.

Table S3. Performance characteristics of the analytical method used.

Figure S1. Bar chart visualizing Ca levels (mean±SD) measured in bee pollen in our study and by other researchers [42-44].

Figure S2. Bar chart visualizing Cu levels (mean±SD) measured in bee pollen in our study and by other researchers [39,40,42-44].

Figure S3. Bar chart visualizing Cr levels (mean±SD) measured in bee pollen in our study and by other researchers [39,40,42].

Figure S4. Bar chart visualizing Fe levels (mean±SD) measured in bee pollen in our study and by other researchers [39-44].

Figure S5. Bar chart visualizing K levels (mean±SD) measured in bee pollen in our study and by other researchers [42-44].

Figure S6 Bar chart visualizing Mg levels (mean±SD) measured in bee pollen in our study and by other researchers [41-44].

Figure S7. Bar chart visualizing Mn levels (mean±SD) measured in bee pollen in our study and by other researchers [39,40,42-44].

Figure S8. Bar chart visualizing Na levels (mean±SD) measured in bee pollen in our study and by other researchers [42-44].

Figure S9. Bar chart visualizing Ni levels (mean±SD) measured in bee pollen in our study and by other researchers [39-42].

Figure S10. Bar chart visualizing Pb levels (mean±SD) measured in bee pollen in our study and by other researchers [39-41].

Figure S11. Bar chart visualizing Zn levels (mean±SD) measured in bee pollen in our study and by other researchers [39-43].

Figure S12. Bar chart visualizing As levels (mean±SD) measured in propolis in our study and by other researchers [45,47,48].

Figure S13. Bar chart visualizing Cr levels (mean±SD) measured in propolis in our study and by other researchers [46,48,49].

Figure S14. Bar chart visualizing Cu levels (mean±SD) measured in propolis in our study and by other researchers [45,47-49].

Figure S15. Bar chart visualizing K levels (mean±SD) measured in propolis in our study and by other researchers [46-48].

Figure S16. Bar chart visualizing Mg levels (mean±SD) measured in propolis in our study and by other researchers [41,46,48].

Figure S17. Bar chart visualizing Pb levels (mean±SD) measured in propolis in our study and by other researchers [41,45-49].

Figure S17. Bar chart visualizing Zn levels (mean±SD) measured in propolis in our study and by other researchers [41,45,46,48,49].

Table S1. Levels (mean±SD) of the selected chemical elements in bee pollen compared to the available literature data.

Element	Matuszewska et al. (2021) - current study	Temizer et al. (2018) [39]	Altunatmaz et al. (2017) [40]	Formicki et al. (2013) [41]	Kostić et al. (2015) [42]	Somerville and Nikol (2002) [43]	Liolios et al. (2019) [44]
**Al**	26.13±20.99	57.79±38.63	-	-	35.87±13.01	-	-
**As**	0.03±0.01	7.03±1.07	0.39±0.43	-	-	-	-
**Ba**	0.64±0.26	-	-	-	1.17±0.94	-	-
**Ca**	1,238.33±539.27	-	-	-	1,448.09±124.81	1146.4±454.51	2,180.00±1,156.00
**Cd**	0.06±0.05	-	0.07±0.04	50.83±21.46	0.07±0.03	-	-
**Co**	0.038±0.012	-	-	-	0.037±0.01	-	-
**Cr**	0.07±0.04	0.37±0.13	0.79±0.37	-	0.23±0.03	-	-
**Cu**	4.75±1.23	4.95±2.09	10.42±3.05	-	7.89±0.66	12.4±10.24	10.00±4.00
**Fe**	49.17±22.16	71.84±33.16	203.17±184.21	1.23±0.22	68.09±8.54	67.16±72.13	109.00±46.00
**K**	4,233.33±398.33	-	-	-	3,482.40±274.26	5530±4,881.34	5,899.00±2,031.00
**Mg**	823.33±235.09	-	-	1,946.25±44	780.76±95.55	716±394.47	962.00±388.00
**Mn**	25.00±18.44	19.59±9.74	29.33±41.56	-	24.15±12.50	32.68±25.15	38.00±34.00
**Mo**	0.23±0.07	0.22±0.25	-	-	-	-	-
**Na**	25.17±6.65	-	-	-	23.16±4.47	82.02±84.96	300.00±139.00
**Ni**	0.65±0.15	0.65±0.34	0.51±0.57	5.71±1.78	0.75±0.30	-	-
**P**	4,050.00±582.24	-	-	-	-	4600±1,672.47	4,325.00±2,053.00
**Pb**	0.15±0.05	0.34±0.33	0.13±0.13	1.67±0.40	-	-	-
**S**	2,383.33±416.73	-	-	-	-	2378±692.91	-
**Se**	0.05±0.03	-	2.56±0.99	-	-	-	-
**Si**	40.25±28.02	-	3.22±3.76		-	-	-
**Zn**	31.33±5.75	17.78±5.18	29.15±6.60	108.66±24.84	41.06±5.55	58.28±47.23	-

Table S2. Levels (mean±SD) of the selected chemical elements in propolis compared to the available literature data.

Element	Matuszewska et al. (2021) - current study	Formicki et al. (2013) [41]	Roman et al. (2011) [45]	Finger et al. (2014) [46]	Hodel et al. (2020) [47]	Bonvehí and Bermejo (2012) [48]	Popov et al. (2017) [49]
**Ag**	0.12±0.10	-	-	-	-	0.058±0.007	-
**Al**	26.13±20.99	-	-	680.00±610.00	-	460.00±62.20	-
**As**	0.03±0.01	-	0.657±0.38	-	2.736±3.79	0.09±0.02	-
**Ca**	1,238.33±539.27	-	-	1,660.00±1070.00	-	3,443.00±1,672.00	-
**Cd**	0.06±0.05	28.24±15.84	0.19±0.18	0.13±0.17	0.03*	0.07±0.02	0.02±0.01
**Co**	0.038±0.012	-	-	-	-	0.29±0.26	-
**Cr**	0.07±0.04	-	-	5.53±3.53	-	1.42±0.72	0.03±0.013
**Cu**	4.75±1.23	-	6.95±4.05	-	4.15±2.85	3.45±0.68	0.023±0.005
**Fe**	49.17±22.16	0.55±0.26	-	-	-	572.00±303.00	-
**K**	4,233.33±398.33	-	-	7,590.00±870.00	1,774.21±1,996.38	2,227.00±779.00	-
**Mg**	823.33±235.09	362.13±202.53	-	1,270.00±640.00	-	814.00±318.00	-
**Mn**	25.00±18.44	-	-	80.00±60.00	-	15.30±7.16	-
**Na**	25.17±6.65	-	-	580.00±400.00	-	159.00±32.60	-
**Ni**	0.65±0.15	4.92±2.60	-	-	-	1.99±1.07	-
**P**	4,050.00±582.24	-	-	-	-	404.00±157.00	-
**Pb**	0.15±0.05	1.68±0.75	5.74±4.49	9.85±24.45	0.30±0.25	1.47±1.12	0.04±0.005
**S**	2,383.33±416.73	-	-	-	-	671.00±236.00	-
**Se**	0.05±0.03	-	-	-	0.33±0.10	0.08±0.01	-
**Si**	40.25±28.02	-	-	-	-	694.00±104.00	-
**Zn**	31.33±5.75	39.84±19.02	48.08±22.43	20.00±10.00	-	779.00±376.00	0.03±0.006

* result from only one sample

Table S3. Performance characteristics of the analytical method used.

Analyte	Working range of the method	Detection limit	Quantification limit	Precision determined using unfortified samples (%RSD)	Precision determined using fortified samples (%RSD)	Recovery (%)
**Ag**	0.20 – 40 μg/l<br>0.020 – 4.0 mg/kg	0.047 μg/l<br>0.0047 mg/kg	0.16 μg/l<br>0.016 mg/kg	ND	3.4	102-105
**Al**	1.0 – 200 μg/l<br>0.10 – 20 mg/kg	0.055 μg/l<br>0.006 mg/kg	0.18 μg/l<br>0.018 mg/kg	17	1.5	83-112
**As**	0.20 – 40 μg/l<br>0.020 – 4.0 mg/kg	0.085 μg/l<br>0.0085 mg/kg	0.29 μg/l<br>0.028 mg/kg	26	5.2	103-118
**Ba**	2.0 – 400 μg/l<br>0.20 – 40 mg/kg	0.84 μg/l<br>0.084 mg/kg	2.8 μg/l<br>0.28 mg/kg	39	2.5	101-110
**Cd**	0.10 – 20 μg/l<br>0.010 – 2.0 mg/kg	0.020 μg/l<br>0.002 mg/kg	0.070 μg/l<br>0.007 mg/kg	12	9.7	102-109
**Co**	0.10 – 20 μg/l<br>0.010 – 2.0 mg/kg	0.015 μg/l<br>0.0015 mg/kg	0.049 μg/l<br>0.0049 mg/kg	32	4.1	103-112
**Cr**	0.20 – 40 μg/l<br>0.020 – 4.0 mg/kg	0.031 μg/l<br>0.003 mg/kg	0.10 μg/l<br>0.01 mg/kg	24	2.4	106-113
**Cu**	1.0 – 200 μg/l<br>0.10 – 20 mg/kg	0.078 μg/l<br>0.0078 mg/kg	0.26 μg/l<br>0.026 mg/kg	22	2.5	102-113
**Mn**	2.0 – 400 μg/l<br>0.20 – 40 mg/kg	0.40 μg/l<br>0.04 mg/kg	1.3 μg/l<br>0.13 mg/kg	19	5.5	94-108
**Ni**	0.20 – 40 μg/l<br>0.020 – 4.0 mg/kg	0.083 μg/l<br>0.0083 mg/kg	0.28 μg/l<br>0.028 mg/kg	23	4.6	102-109
**Pb**	0.20 – 40 μg/l<br>0.020 – 4.0 mg/kg	0.010 μg/l<br>0.001 mg/kg	0.030 μg/l<br>0.003 mg/kg	25	1.0	100-104
**Se**	0.20 – 40 μg/l<br>0.020 – 4.0 mg/kg	0.17 μg/l<br>0.017 mg/kg	0.56 μg/l<br>0.056 mg/kg	ND	12	106-138
**V**	0.20 – 40 μg/l<br>0.020 – 4.0 mg/kg	0.050 μg/l<br>0.005 mg/kg	0.17 μg/l<br>0.017 mg/kg	22	5.2	104-111
**Zn**	1.0 – 200 μg/l<br>0.10 – 20 mg/kg	0.54 μg/l<br>0.054 mg/kg	1.8 μg/l<br>0.18 mg/kg	38	2.3	77-162

<QL – below quantification limit

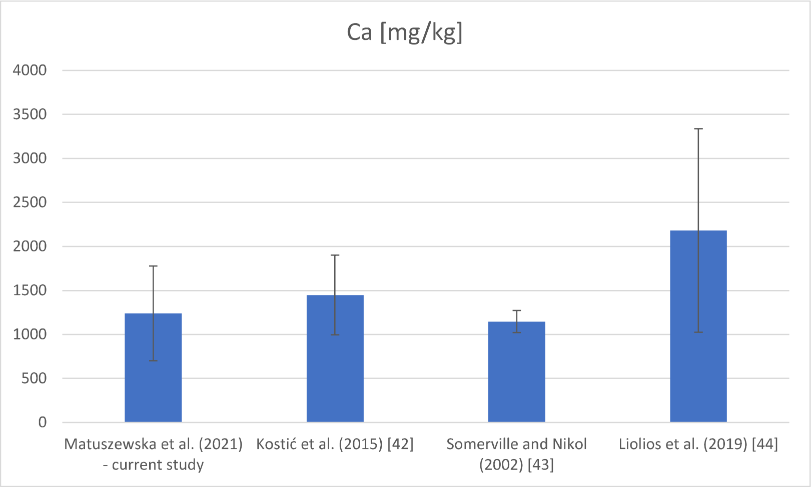

Figure S1. Bar chart visualizing Ca levels (mean±SD) measured in bee pollen in our study and by other researchers [42-44].

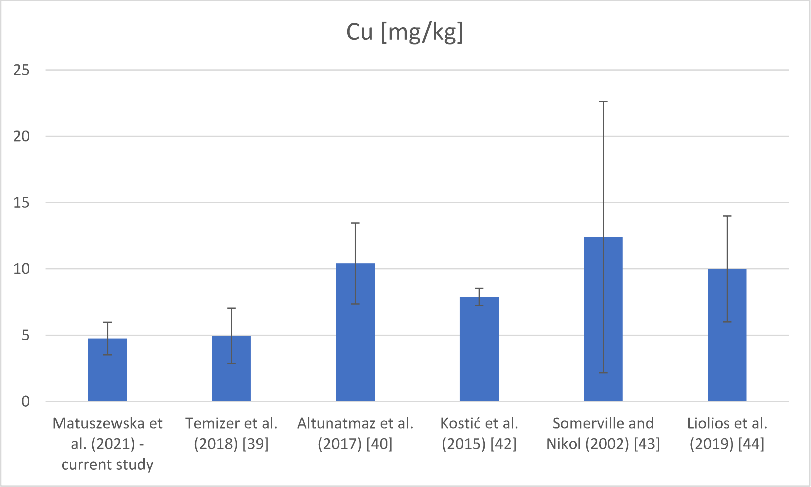

Figure S2. Bar chart visualizing Cu levels (mean±SD) measured in bee pollen in our study and by other researchers [39,40,42-44].

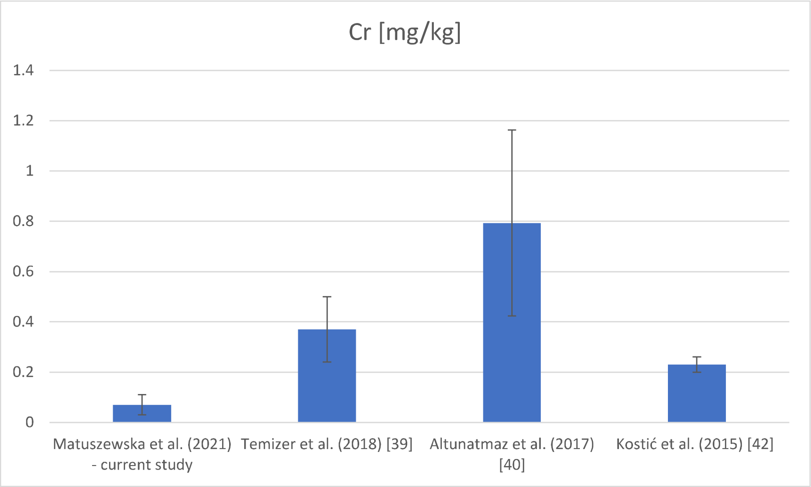

Figure S3. Bar chart visualizing Cr levels (mean±SD) measured in bee pollen in our study and by other researchers [39,40,42].

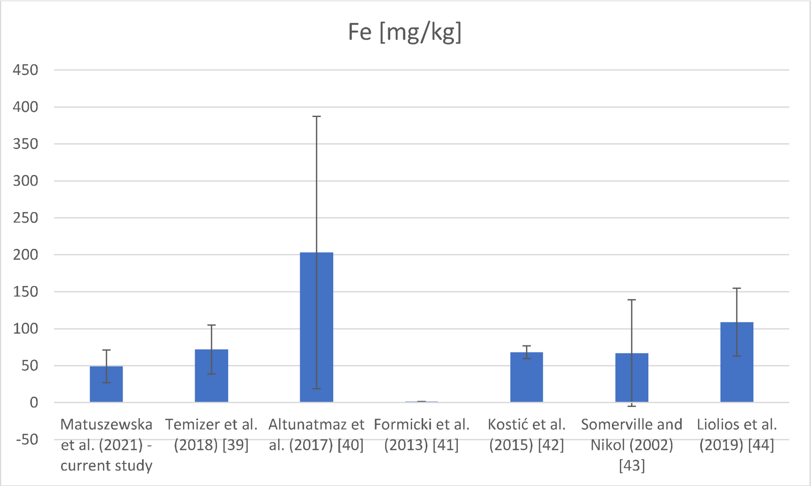

Figure S4. Bar chart visualizing Fe levels (mean±SD) measured in bee pollen in our study and by other researchers [39-44].

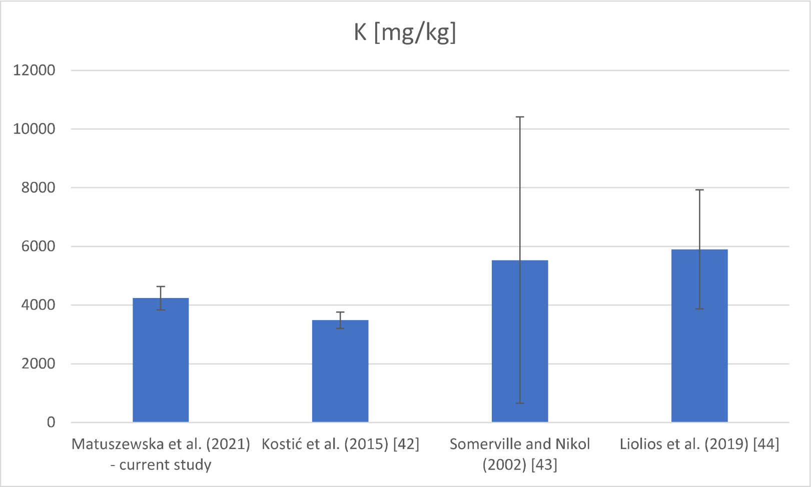

Figure S5. Bar chart visualizing K levels (mean±SD) measured in bee pollen in our study and by other researchers [42-44].

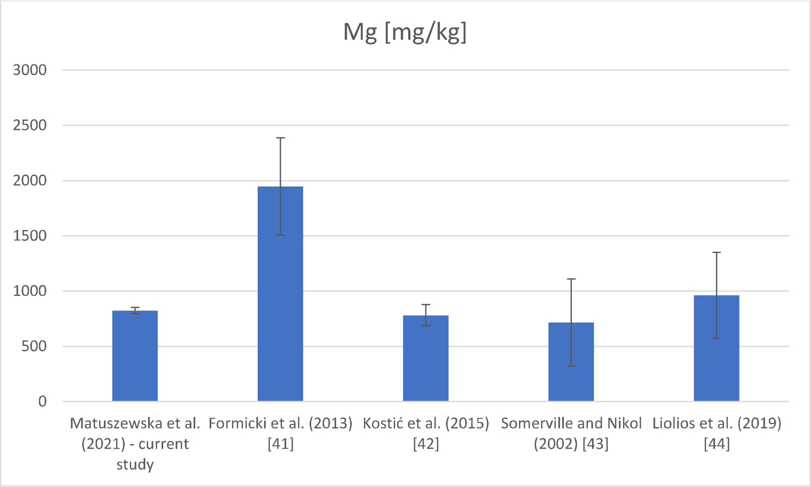

Figure S6 Bar chart visualizing Mg levels (mean±SD) measured in bee pollen in our study and by other researchers [41-44].

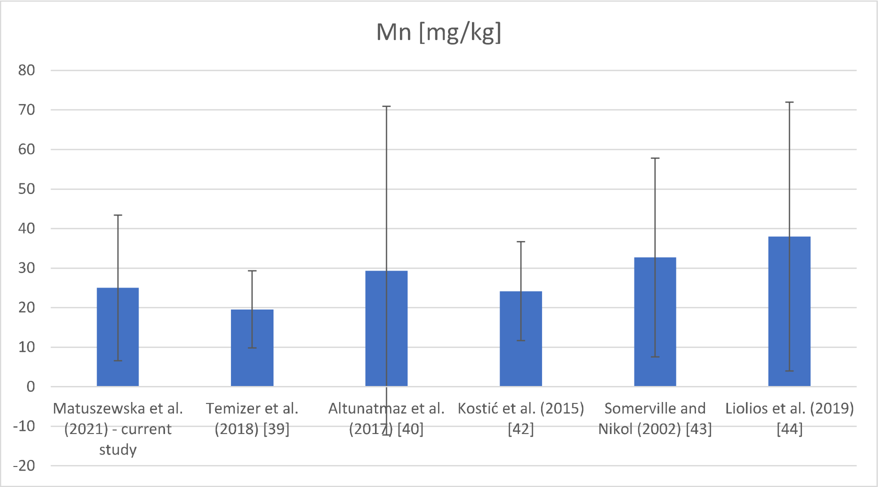

Figure S7. Bar chart visualizing Mn levels (mean±SD) measured in bee pollen in our study and by other researchers [39,40,42-44].

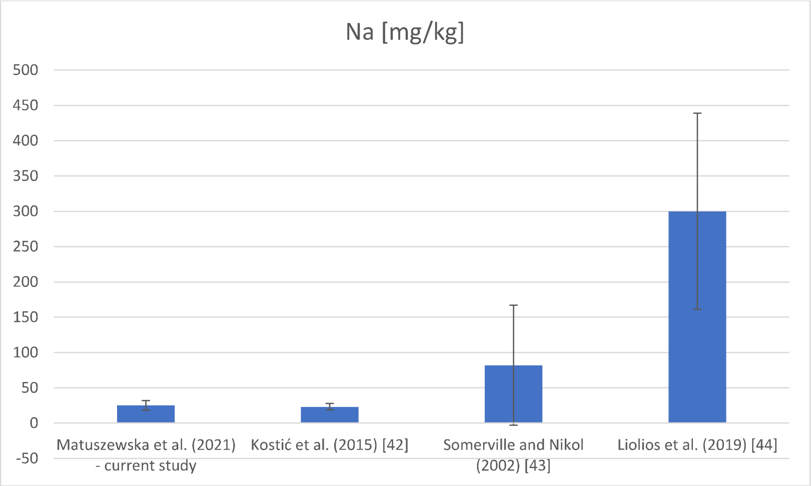

Figure S8. Bar chart visualizing Na levels (mean±SD) measured in bee pollen in our study and by other researchers [42-44].

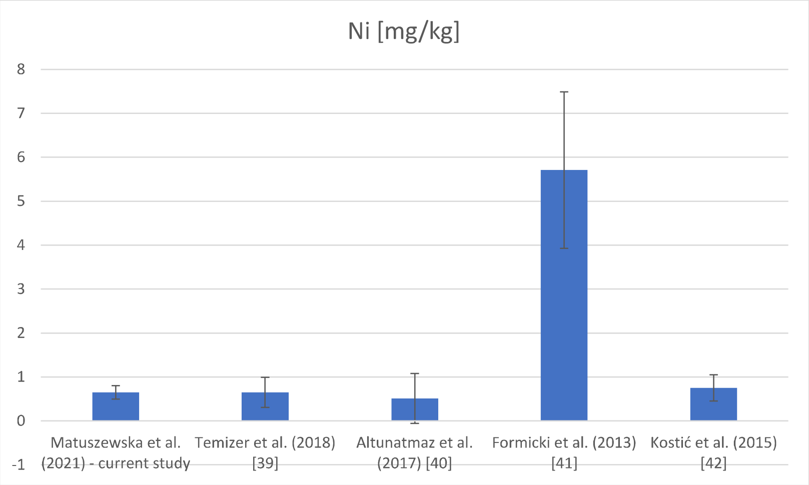

Figure S9. Bar chart visualizing Ni levels (mean±SD) measured in bee pollen in our study and by other researchers [39-42].

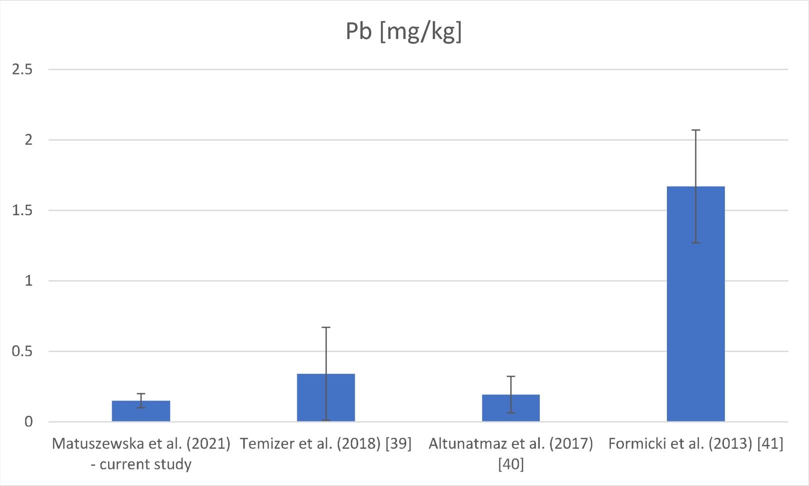

Figure S10. Bar chart visualizing Pb levels (mean±SD) measured in bee pollen in our study and by other researchers [39-41].

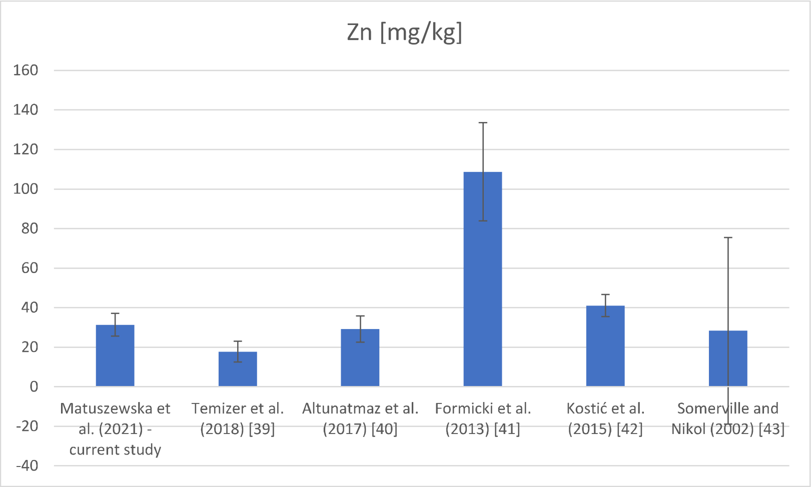

Figure S11. Bar chart visualizing Zn levels (mean±SD) measured in bee pollen in our study and by other researchers [39-43].

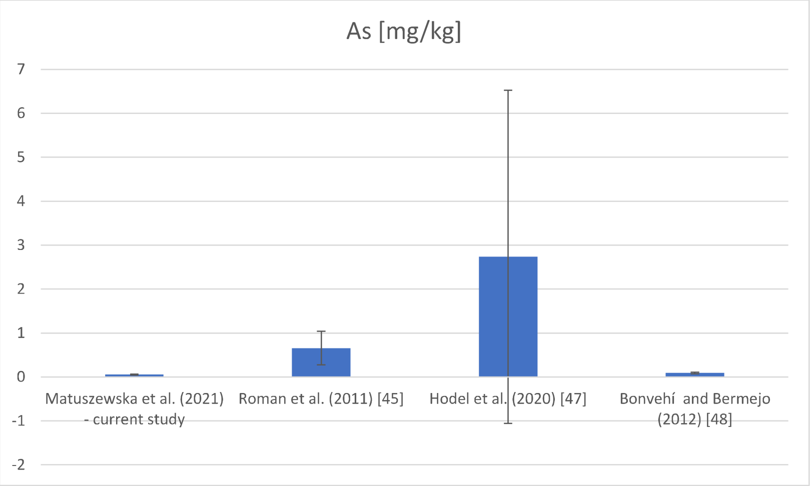

Figure S12. Bar chart visualizing As levels (mean±SD) measured in propolis in our study and by other researchers [45,47,48].

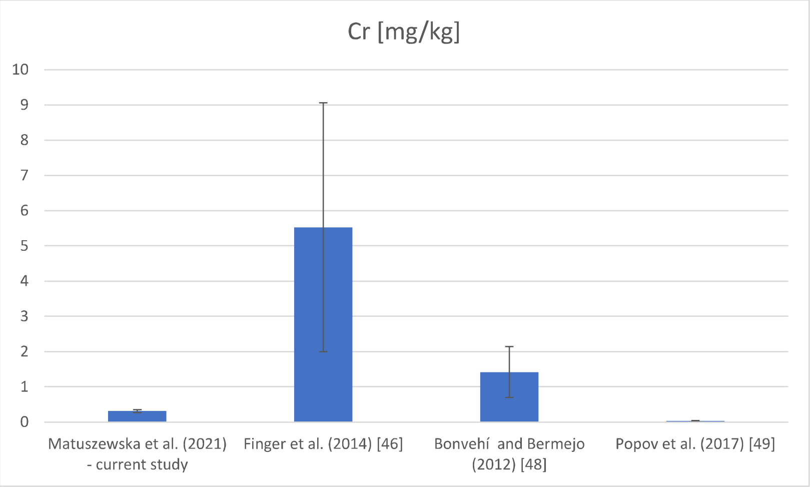

Figure S13. Bar chart visualizing Cr levels (mean±SD) measured in propolis in our study and by other researchers [46,48,49].

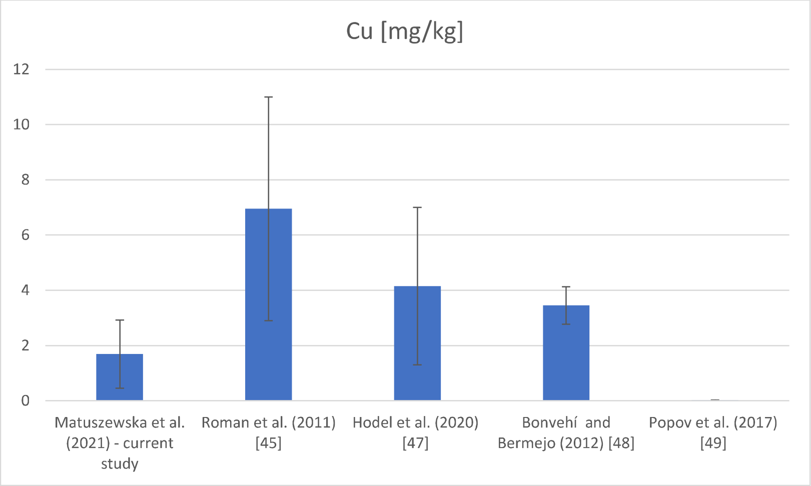

Figure S14. Bar chart visualizing Cu levels (mean±SD) measured in propolis in our study and by other researchers [45,47-49].

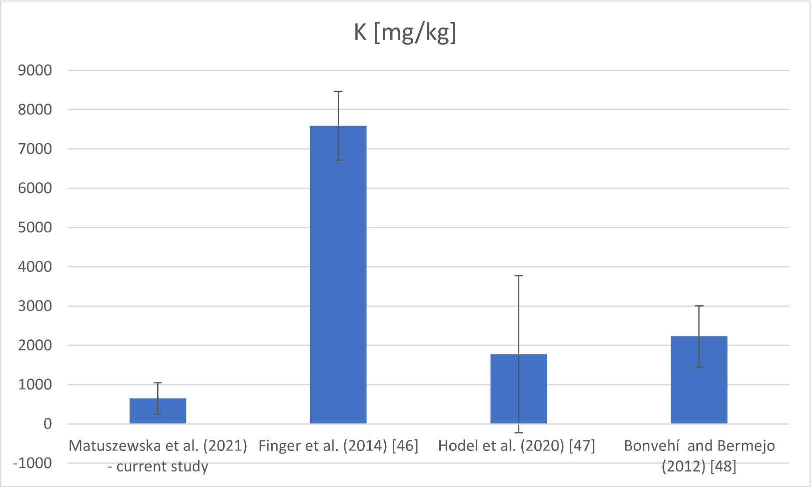

Figure S15. Bar chart visualizing K levels (mean±SD) measured in propolis in our study and by other researchers [46-48].

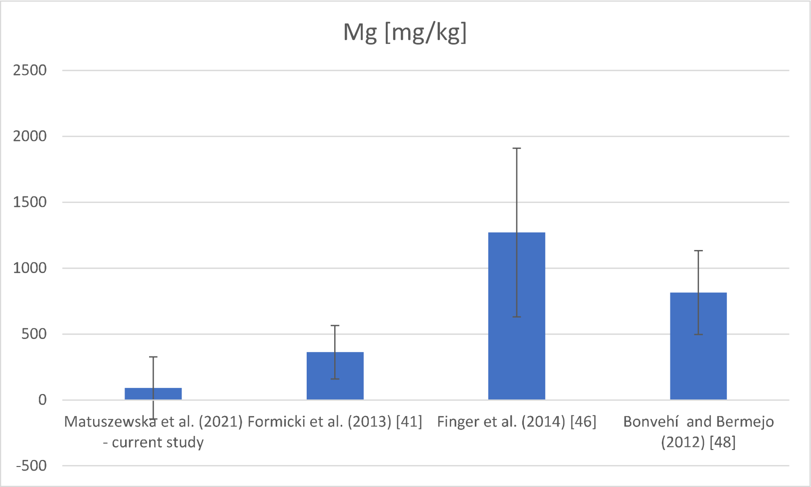

Figure S16. Bar chart visualizing Mg levels (mean±SD) measured in propolis in our study and by other researchers [41,46,48].

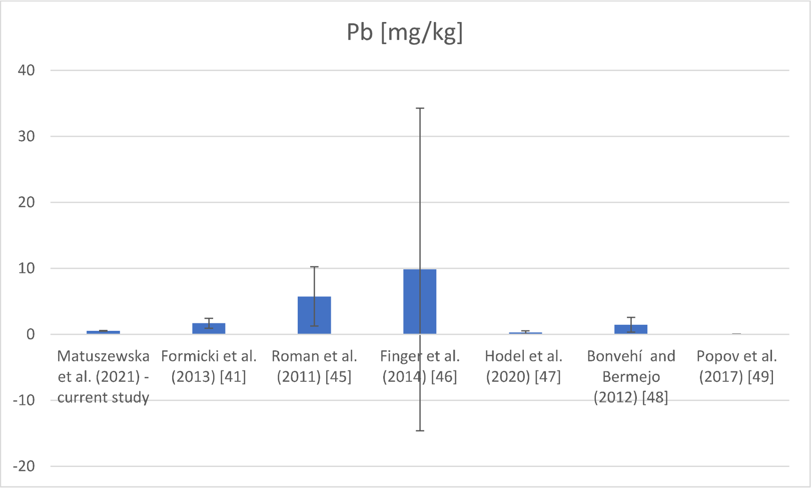

Figure S17. Bar chart visualizing Pb levels (mean±SD) measured in propolis in our study and by other researchers [41,45-49].

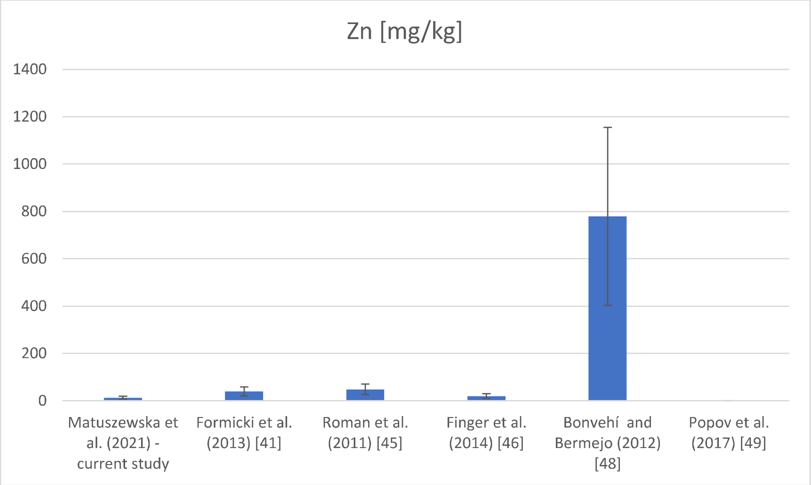

Figure S17. Bar chart visualizing Zn levels (mean±SD) measured in propolis in our study and by other researchers [41,45,46,48,49].

References:

39. Temizer İK, Güder A, Temel FA, AVCI E. A comparison of the antioxidant activities and biomonitoring of heavy metals by pollen in the urban environments. *Environ Monit Assess* **2018**;190(8):462.
40. Altunatmaz SS, Tarhan D, Aksu F, Barutçu UB, Or ME. Mineral element and heavy metal (Cadmium, lead and arsenic) levels of bee pollen in Turkey. *Food Sci Technol* **2017**;37:136–41.
41. Formicki, Grzegorz; Greń, Agnieszka; Stawarz, Robert; Zyśk, Bartłomiej; Gał A. Metal Content in Honey, Propolis, Wax, and Bee Pollen and Implications for Metal Pollution Monitoring. *Polish J Environ Stud* **2013**;22(1):99–106.
42. Kostić AZ, Pešić MB, Mosić MD, Dojèinović BP, Natić MM, Trifković JD. Mineral content of bee pollen from Serbia. *Arh Hig Rada Toksikol* **2015**;66(4):251–8.
43. Somerville DC, Nicol HI. Mineral content of honeybee-collected pollen from southern New South Wales. *Aust J Exp Agric* **2002**;42(8):1131–6. https://doi.org/10.1071/EA01086
44. Liolios V, Tananaki C, Papaioannou A, Kanelis D, Rodopoulou MA, Argena N. Mineral content in monofloral bee pollen: investigation of the effect of the botanical and geographical origin. *J Food Meas Charact* **2019**;13(3):1674–82. http://dx.doi.org/10.1007/s11694-019-00084-w
45. Roman A, Madras-Majewska B, Popiela-Pleban E. Comparative study of selected toxic elements in propolis and honey. *J Apic Sci* **2011**;55(2):97–106.
46. Finger D, Filho IK, Torres YR, Quináia SP. Propolis as an indicator of environmental contamination by metals. *Bull Environ Contam Toxicol* **2014**;92(3):259–64.
47. Hodel KVS, Machado BAS, Santos NR, Costa RG, Menezes-Filho JA, Umsza-Guez MA. Metal Content of Nutritional and Toxic Value in Different Types of Brazilian Propolis. von Muhlen C, editor. *Sci World J* **2020**;2020:4395496.
48. Bonvehí JS, Bermejo FJO. Element content of propolis collected from different areas of South Spain. *Environ Monit Assess* **2013**;185(7):6035–47. https://doi.org/10.1007/s10661-012-3004-3
49. Popov BB, Hristova VK, Presilski S, Shariati MA, Najman S. Assessment of heavy metals in propolis and soil from the Pelagonia region, republic of Macedonia. *Maced J Chem Chem Eng* **2017**;36(1):1–11.
